# Unsuspected *Strongyloides stercoralis* infection in hospital patients with comorbidity in need of proper management

**DOI:** 10.1186/s12879-016-1424-3

**Published:** 2016-02-29

**Authors:** Rina Lisette Girard Kaminsky, Selvin Zacarías Reyes-García, Lysien Ivania Zambrano

**Affiliations:** Department of Pediatric, School of Medical Sciences, National Autonomous University of Honduras, and Parasitology Service, Department of Clinical Laboratory, University Hospital, Tegucigalpa, Honduras; Department of Morphological Sciences, School of Medical Sciences, National Autonomous University of Honduras, Tegucigalpa, Honduras

**Keywords:** Eosinophilia, HIV/AIDS, Honduras, Immunocompromised host, Strongyloidiasis, Strongyloides stercoralis

## Abstract

**Background:**

Investigate the role of latent strongyloidiasis infection in patients at the University Hospital, Honduras.

**Methods:**

Prospective observational cohort study during 20 non consecutive months from March 2009 to February 2011. Epidemiological and clinical data obtained from patients excreting *Strongyloides stercoralis* larvae in stool who consulted at the hospital were recorded and analyzed.

**Results:**

Thirty five (5 %) of 712 patients had *S. stercoralis* larvae in one stool sample; 62.8 % came from rural areas and 91.7 % were poor; 68.5 % (24/35) were 21 years old or older. Eight patients (22.8 %) had no predisposing illness; 3 (8.6 %) received steroid treatment, 29/35 (82.8 %) presented with persistent diarrhea and 24/35 (68.5 %) presented following comorbidities: HIV/AIDS (31.4 %), alcoholism alone (11.4 %) or with other associated illness (8.6 %), malignancy (8.6 %), renal failure (5.7 %) and hyperthyroidism (2.8 %). A combination of symptoms suggestive of strongyloidiasis but indistinguishable from those potentially associated to their comorbid condition included severe epigastric pain, diarrhea of weeks duration, peripheral eosinophilia, astenia, adynamia, fever, anemia and weight loss in 85.7 % of the cases, 3 of whom described skin lesions compatible with larva currens. None of the diagnostic clinical impressions mentioned *Strongyloides* infection. Ten strongyloidiasis patients received partial treatment with albendazole or ivermectin. Incomplete data, underestimation of the parasitic infection and no laboratory follow-up of the patients limited our observations.

**Conclusions:**

*Strongyloides stercoralis* is an unsuspected and neglected parasitic infection by health personnel in Honduras. Lack of awareness of its importance represents a strong barrier to proper treatment and follow-up, posing a threat of possible fatal complications in patients with comorbid conditions.

## Background

In the last decade interest in *Strongyloides stercoralis* infection and the disease complications it causes has grown globally [1–3]. The parasite is endemic in tropical and some temperate countries in the world where human fecal contamination of the soil is frequent; however, its prevalence has recently been estimated to be 370 million infections worldwide [4]. In a recent systematic review of strongyloidiasis publications, Schär et al. [5]. confirmed that the distribution of this parasitosis is heterogeneous and endemic in many regions of the world. In Latin America and the Caribbean, strongyloidiasis ranged from 1 % in Haiti to 75 % in Perú. Several publications [5, 6] stated that there is little information on *S. stercoralis* distribution or its disease burden in Africa, in part because of the difficulty in diagnosing this infection. Data on its incidence is almost non-existent; a study on strongyloidiasis in 292 randomly selected individuals in Côte d’Ivore [7] found low prevalence rates among children but high in adults, and questioned the possibility of a higher incidence if in fact many adult patients acquired the infection during childhood. High rates of infection have been recorded in African refugee populations. A study conducted in the United States found that 46 % of 462 Sudanese and 23 % of 100 Somali Bantu refugees were infected with *S. stercoralis* [8]. This highlights potential hazards in the transportation of this parasite across borders in the current climate of mass population movements [9]. The implementation of more rigorous diagnostic methods and properly trained and supervised technicians may improve results, as shown in a prevalence study on *S. stercoralis* infection in rural Cambodia utilizing the Koga agar plate culture method (sensitivity 81.9 %) and a Baermann method (sensitivity 76.8 %); almost every second of 2396 individuals was infected, with a total of 44.7 % infections. An increase of cases, most south of the Yangtze river has been observed in China since the first case was reported in 1973; case reports increased to 330 cases from 1985 to 2011 [10, 11]. Among Indigenous people, rural and remote communities have been found to be highly affected, in particular children and immunocompromised patients [12]. In the Central America region studies are scant or difficult to retrieve; one publication from 1985 reports on eight autopsy cases of individuals who had died with disseminated strongyloidiasis from Costa Rica [13]; three were children and five adults; also five were females and the clinical diagnoses at admission included two cases of malnutrition with diarrhea, one glomerulonephritis, one chronic alcoholism with hepatic cirrhosis, one chronic nephropathy, one intestinal occlusion, one appendicitis and one colelithiasis with meningioma. Several studies in different selected groups from Honduras were available for review [14]: utilizing the Baermann technique *Strongyloides* larvae were found in 14.7 % of 88 female sex workers, in 3 % of 105 individuals consulting at a health center, in 13.2 % of 106 children < 10 year old from marginal barrios of Tegucigalpa, and in 23.5 % of 79 AIDS patients at a hospital in the north coast of Honduras. Higher percentages of infection were recognized in institutionalized individuals, both children (25.3 % of 99 participants) and adults (25 % of 74 individuals).

The exceptional biologic capacity of *S. stercoralis* to re-infect the human host by a process of auto-infection results in chronic prolonged intestinal infections, some up to 50–75 years after initial contact with the parasite [15]. Although intestinal strongyloidiasis is benign and asymptomatic in most chronic infections in individuals from endemic areas with intact immune systems, in patients presenting risk factors or diseases affecting the immune system the parasite may undergo frequent replications resulting in large numbers of larvae in the intestine and lungs causing hyper-infection or dissemination syndromes with serious consequences to the host as reported in multiple studies from developed non-endemic countries [16–18]. Common symptoms of uncomplicated *S. stercoralis* intestinal infection in endemic areas include epigastric pain of varied intensity, with or without diarrhea, pulmonary symptoms such as cough and wheezing and an urticating rash in case of larva currens due to auto-infection [18, 19]. High-risk patients with malignancies such as haematologic neoplasias and biliary cancer, those receiving corticosteroid treatment [20] or with autoimmune disorders [21], or infected with the human T-lymphotropic virus HTLV-1 [22] may develop a potentially fatal dissemination of the parasite.

The diagnosis of strongyloidiasis depends on finding the larvae in routine fecal examinations, which are very insensitive in light infections when examining only one specimen per patient. Repeated stool samples at different time intervals and the use of more sensitive methods of larval extraction such as Baermann and agar migration or Koga method yield better results, but are seldom used in public health facilities due to time, cost and demand of better trained laboratory personnel [23, 24]. The introduction of screening tests such as ELISA or molecular techniques highly sensitive as a diagnostic tool for parasitic infections such as DNA detection using conventional or real time PCR promise better alternatives once certain difficulties are overcome such as cost and the requirement of better public health laboratory infrastructure in developing countries [25, 26].

Previous investigations in selected groups in Honduras utilizing the Baermann method disclosed high *Strongyloides* infection in children (25/99, 25.3 %) living in an institution and institutionalized adults (18/74, 25 %) being treated for mental disorders and/or alcoholism; in patients at a health facility (70/427, 17 %) and in AIDS (15/80, 18.7 % to 12/56, 21.4 %) patients; however, the type of illness, patient complaints and signs and symptoms remained largely undefined [14]. A *Strongyloides* hyperinfection case in a Honduran woman with AIDS [27] raised the question whether similar cases were being missed for lack of suspicion or awareness. Results on strongyloidiasis from the studies mentioned in this paragraph represented unsuspected new findings; also the information at hand was related to the proportion of strongyloidiasis in individuals diagnosed during stool examinations by the modified Baermann method and who consulted at a hospital and/or were institutionalized. In view of those results and because strongyloidiasis is rarely suspected, it was considered of interest to determine whether individuals infected with *S. stercoralis* larvae presented with any particular clinical profile which description could guide the clinical diagnosis or disease perception during ambulatory consultation or internment at the study site. Therefore, the aim of this study was to investigate the role of latent and unsuspected strongyloidiasis infection in patients attending the University Hospital; signal to its potential risk for serious complications in individuals with comorbid conditions and to contribute to strongyloidiasis data in the region. Here we present results on the socio-demographic characteristics of the study group, main signs and symptoms and associations found between *Strongyloides* infection and other comorbid illnesses.

## Methods

### Study site

This study was conducted through the Parasitology Service (PS), Department of Clinical Laboratory of the University Hospital in the capital city Tegucigalpa, Honduras, Central America. Honduras has the highest levels of economic inequality, 64.5 % of the population live in poverty, with 42.6 % living in extreme poverty. Six of each 10 households in the rural area live underneath the poverty line. (World Bank Sept. 2015) The UH is a tertiary level care facility with a capacity of about 1050 hospital beds attending mostly a poor or below poverty line population, serving also as a referral center for the country and as a teaching facility for the adjacent School of Medicine of the National Autonomous University of Honduras. It is divided into a Maternal and Child and a Medico-Surgical hospitals, respectively, providing medical emergency and ambulatory care for individuals of all ages from low economic class through several specialty clinics, in-patient care and surgery. The PS provides routine diagnosis of parasitic infections by experienced technicians and two parasitology specialists, for both intestinal and vector transmitted parasites, during the morning shift from 7:00 a.m. to 2:00 p.m. five days a week; two other shifts were not included since they are conducted outside the PS quality control supervision. The PS receives an average of 4,000 stool specimens and 600 blood samples a year. This investigation was descriptive and non interventional in nature, during 20 non-consecutive months starting March 2009 to February 2011. The unplanned interruptions during the study were due to time availability, vacations, holidays and other involuntary logistical constrains during teaching responsibilities (RGK).

### Ethics Committee

The Ethics Committee of the School of Medical Sciences at the National Autonomous University of Honduras approved the methodology and the study since its modality posed no risk to the patients. Both the Chief of Pediatric and the Chief of Internal Medicine wards from the UH were informed of the purpose of the study and granted consent to review of medical histories of the strongyloidiasis patients. The patients identified with *S. stercoralis* infection and the parents or guardians of the infected minors were contacted and explained the purpose of the study; each provided a written consent for granting participation in an interview and to extract pertinent socio-demographic and clinical data from their respective clinical files, respecting confidentiality.

### Financing support

This investigation was conducted as part of research responsibilities at the School of Medical Sciences, Department of Pediatric and did not receive any financial support, besides regular salary, from any institution or organization.

### Population studied

The definition of strongyloidiasis in an individual was based on the identification of *S. stercoralis* larvae in his/her stool sample. Infected individuals were identified during the daily routine direct stool examinations. Once *S. stercoralis* larvae were identified, pertinent data of name, sex, age, and the remitting clinic in the hospital were entered manually in the participant case report form; the patient was contacted and interviewed as soon as possible. Demographic data included socio-economic status, place of residence, family size, sanitary facilities of toilet use, water and garbage disposal, alcoholism, and whether living in an institution. Reason for consultation and clinical data were taken from the clinical history such as signs, symptoms, duration of present complaints, associated disease and hematology laboratory results. When the patient at ambulatory care was released before issuing laboratory results the responsible physician was personally advised of the case. No negative controls were included for comparison of epidemiological or clinical characteristics.

### Laboratory methodology

The routine methodology at the PS includes following procedures: a macroscopic examination to determine stool consistency, presence of mucus and/or blood, and other adult parasites or segments; a microscopic examination of a direct 2 mg suspension of feces in physiologic saline and a Lugol suspension, respectively; helminth egg count when necessary, to estimate the intensity of the infection; samples from children 0 to 5 years old or from HIV/AIDS patients as requested by the physician are routinely stained with a modified Ziehl-Neelsen carbol-fuchsin (ZNM) technique for identification of intestinal apicomplexa. For the present investigation, when no *S. stercoralis* larvae were detected during the direct examinations in physiologic saline and Lugol solution, 4 to 6 stools were selected at random from the daily workload and examined by a modified Baermann method and/or an agar migration method (Koga), both described below. For the Baermann method [28], about 5 g of stool were spread on a double-layered piece of gauze or the whole stool sample when the amount delivered by the patient was very small. This preparation was introduced in a 250 mL sedimentation glass previously filled with water, and left minimum one hour before retrieving at least 3 mL of the sediment with a Pasteur pipette, and placed in a small Petri dish. The sediment was then examined with a stereoscopic microscope. When positive for larvae, a few were aspirated from the Petri dish, placed between slide and cover slip with a drop of Lugol and examined under an optical microscope to differentiate the larvae by morphology. For the Koga method, 2–3 g of stool were carefully spread on the surface of the agar plate (1.5 % agar, 0.5 % beef extract, 1.0 % peptone and 0.5 % sodium chloride) and observed under a stereoscopic microscope after 24 h incubation at 28 °C [28]. Whether positive for migrating larvae or not, each agar plate was washed with 3 mL 10 % formalin solution, centrifuged and the sediment searched for larvae under an optical microscope as stated before. Other incidental parasitologic findings were registered as well.

## Results

During the 20 months’ study duration a total of 712 stool samples were examined by the direct method; of those, 300 (42.1 %) were examined additionally by the Baermann technique and 100 (14.0 %) by the Koga method (Table 1). The direct method identified 25 individuals, eight samples were positive for Baermann and a total of eight positives were found by the Koga method, of which 4 had been recovered in the Baermann sediment for a total of 35 *Strongyloides* infections. The stool examination requests were distributed as follows: 309 (43.4 %) from adult ambulatory care, 101 (14.1 %) from adult wards, 207 (29.0 %) from pediatric ambulatory care, 48 (6.7 %) from pediatric wards and 47 (6.6 %) from pediatric oncology ambulatory care and ward. There was a higher proportion of *Strongyloides* infections in individuals from the adult wards (14/101, 13.8 %) and the pediatric wards (5/48, 10.4 %; ×^2^ = 31.381; *p* = 0.01). Other mixed parasitic infections (Table 1) included 3 hookworm infections, two severe with 87 and 2,388 eggs in 2 mg stool suspension, all except two of 15 *Trichuris* infections were light and three of the 15 *Ascaris lumbricoides* infections were severe, with more than 100 eggs per direct smear (2 mg stool suspension); *Cystoisospora belli* oocysts were found in repeated stool examinations in an AIDS patient.

**Table 1:** Total stools/total examined for *Strongyloides* larvae, ambulatory care and wards; mixed intestinal parasitic infections, University Hospital, 2009-2012, Honduras

Parameter	Number (%)	Intestinal parasites in 712 patients, ambulatory care and wards (%)						
		A.l.	T.t.	S.s.	Eh/Ed	G.d.	C.c.	C. spp
Total received/Total examined for *Strongyloides*	46,616/712 (1.5)							
Direct method	712 (100)	15	15	25 (3.7)	24	24		
Baermann	300 (42.1)			8 (2.6)				
Agar migration	100 (14.0)			8 (8.0)				
ZNM							8	3
Ambulatory care adults	309 (43.4)	2	1	12	11	6	3	2
Adult wards	101 (14.1)	3	4	14*	3	1	0	1
Ambulatory care pediatrics	207 (29.0)	4	2	3	6	9	5	0
Pediatric wards	48 (6.7)	3	2	5*	0	2	0	0
Pediatric oncology	47 (6.6)	3	3	1	2	6	0	0

Abbreviations: % percentaje, *ZNM* Ziehl-Neelsen modified method, *A.l. Ascaris lumbricoides*, *T.t. Trichuris trichiura*, *S.s. Strongyloides stercoralis*, *Eh*/*Ed Entamoeba histolytica*/*E. dispar*, *G.d. Giardia duodenalis*, *C.c. Cyclospora cayetanensis*, *C.spp. Cryptosporidium* spp. *p < 0.01 (×^2^ = 31.381)

Demographic data of the 35 infected patients are shown in Table 2. Of the 35 positive S. *stercoralis* individuals, 29 (82.8 %) reported a history of diarrhea of weeks or months duration and many daily episodes; however, only 13 of the 24 stool samples were diarrheic or liquid when delivered to the PS for examination; in 9 cases the stool consistency was not registered. No other exams such as sputum and duodenal aspiration were requested from any of the patients.

**Table 2:** Socio-demographic characteristics in 35 individuals infected with S*trongyloides stercoralis*, University Hospital, Honduras

Characteristics	Number (%)
Total	35
Sex	
Men	18 (51.4)
Women	17 (48.5)
Age range (years)	
0-10	4 (11.4)
11-20	8 (22.8)
21-35	11 (31.4)
36-49	5 (14.2)
≥50	8 (22.8)
Residence	
Rural	22 (62.8)
Urbano-marginal	10 (28.5)
Not registered	3 (8.6)
Schooling	
No school	8 (22.8)
Incomplete primary	10 (28.5)
Secundary	1 (2.8)
University	2 (5.7)
Not registered	14 (40.0)
Hygiene	
No letrine	4 (11.4)
Letrine	12 (34.2)
Washable toilet	2 (5.7)
No information	17 (48.6)
Average shared living	6 persons
Range	1-14 children & adults

The clinical findings in the 35 patients with strongyloidiasis are outlined in Table 3. Alcoholism in patients was defined as a dependence or excessive use of alcohol, some of whom reported drinking since adolescence. The most common complaint was diarrhea in 29 (82.8 %) individuals, of weeks or months duration but without defining its characteristics (Table 4). The weight loss reported was between 5 and 38 pounds in 13 cases; 15 (42.8 %) patients reported anemia with an hemoglobin range between 2.5 and 10.8 vol.%; 15 out of 24 patients had eosinophilia (using % of the total WBCs) (range 5.4 % -63.4 %), with an average of 14.7 %; no eosinophilia data were available in 11 cases. There were only two documented immunology reports of IgE levels: a 16 years old with no comorbidity and an 11 month old child being treated with steroids “because of atopy” with IgE levels of 3112 mg/dL and 3500 mg/dL, respectively (normal range 720–1560 mg/dL). Several clinical histories had incomplete data of different kind. Eight (22.8 %) of the 35 strongyloidiasis infected patients had other parasitic infections: *Trichuris trichiura*, human hookworm and *Giardia duodenalis*; *Blastocystis* spp.; *T. trichiura*, *Balantidium coli*, and human hookworm; *Cystoisospora belli*; human hookworm; *Trichomonas hominis* and *Blastocystis* spp.; one case with cerebral toxoplasmosis and one patient with *Entamoeba coli*, *Endolimax nana* and *Blastocystis* spp infections. Only in 10 cases treatment against strongyloidiasis was mentioned, 8 with albendazol and 2 with ivermectin, which were partially administered in the hospital before medical release; follow up was not possible, patients were not controlled for strongyloidiasis cure before leaving the hospital. Human hookworm and *Trichuris* infections were treated with mebendazole without follow-up; cerebral toxoplasmosis was treated with unkown results. The *C. belli* infection was treated with trimetroprim-sulfametoxazole. Four patients died as shown in Table 4.

**Table 3:** Clinical features of 35 individuals with associated illnesses and *Strongyloides stercoralis* larvae in stools, Honduras

No. of patients	Co-morbidity/Risk factor	Signs & Symptoms
8	None detected	Diarrhea, severe abdominal pain, adynamia, asthenia, weight loss, eosinophilia, pruritic skin lesions in 2 patients
4	Alcoholism	Diarrhea, severe epigastric pain, fever, adynamia, asthenia, weight loss, 2 patients lived in an institution
2	Malignancy	Diarrhea, fever, anemia, adynamia, weight loss, asthenia, eosinophilia, vomiting
11	HIV/AIDS^a^	Diarrhea, abdominal pain, adynamia, asthenia, weight loss, anemia, fever, alcoholism in 3, intense gluteal pruritus in 1 patient, cerebral toxoplasmosis in one, eosinophilia in one, 3 lived in an institution
3	Asthma, steroid treatment	Diarrhea, severe abdominal pain in 1 patient; cough, dermatitis in 1 patient; scalp mycosis in 1 patient with 63.4 % eosinophilia
2	Renal failure	Asthenia, adynamia, anemia, melena in 1 patient, dyspnea, weight loss
1	Gluteal abscess	Fever, eosinophilia
1	Cerebral palsy	Diarrhea, abdominal pain, fever, anemia, severe hhkw & *Trichuris* infections
1	Hypertension, cardiopathy	No other clinical data in the history, except patient living in institution
1	Obesity	Admitted for severe abdominal pain, eosinophilia, cough, insomnia
1	Cough, fever	No other data registered

^a^ *HIV*/*AIDS* human immunodeficiency virus/acquired immune-deficiency syndrome, *hhkw* human hookworm infection

**Table 4:** Distribution and characteristics of eosinophilia in 15/24 (62.5 %) patients with strongyloidiasis, Honduras

Percentage eosinophils	Age years	Relevant data
5.4	34	AIDS, alcoholism, COPD, suspected peritoneal TB, died
6.8	43	Hyperthyroidism, alcoholism
8.2	59	Ulcer in left foot, fever, anemia
9.6	78	Intestinal carcinoma suspected, died
6.7-13.8	29	Anemia, general weakness
10.8	9	Severe intestinal parasitism, severe anemia
12.6	11	Mielocytic leukemia, died
13.2	17	Gluteal abscess, fever, improves with antibiotics
30.5	65	Anemia, severe intestinal parasitism, died
38.9	38	AIDS
50	52	Diarrhea, fever, anemia, intense pruritus gluteal region
59	16	Elevated IgE, gastrointestinal complaints, larvae numerous in stool
63.4	11	Scalp mycosis, steroids

*COPD* chronic obstructive pulmonary disease

## Discussion

This cohort study was carried out on a highly selected population, thus the information obtained is skewed. From the start our primary objective was to identify strongyloidiasis in patients with or without underlying comorbidities and not necessarily to determine frequency or prevalence of this parasite in the participating population; therefore, results of this study are not comparable to similar studies where the main objective was prevalence determination. In the past we observed that in Honduras associations with *S. stercoralis* infections included HIV/AIDS patients, individuals living in an institution, patients with chronic alcoholism, patients with high eosinophilia with no other probable causes and household sharing with an infected person [14]. Many other predisposing factors have been reported to be associated with *Strongyloides* infection, such as malignancy, tuberculosis, transplants, travelers to endemic countries, chronic malnutrition and chronic renal failure [29]. Although a parasitic infection may have been suspected by the clinician in charge, none of the positive cases carried a request for specific *Strongyloides* examination, despite the fact that several had identifiable signs and symptoms of a potential infection with this parasite. Nonetheless, the results confirmed that strongyloidiasis was present and detectable in alcoholic patients and AIDS patients living alone or in an institution; as well as others consulting at this UH with different underlying illnesses such as malignancy, asthma treated with steroids, persistent diarrhea and fever alone or in combination with other signs and symptoms.

A very important finding in this study was the high percentage (82.8 %) of persistent diarrhea reported by 29/35 patients or consigned in their clinical history. Besides parasites, no other bacterial or viral causes were investigated. The diarrhea was not characterized in duration or daily frequency of bowel movements; stool characteristics related only to those observed in one routine sample received in the PS. Although the spectrum of intestinal enteropathy commonly affecting people living in developing countries are multifactorial, the patient epidemiologic history and relevant clinical findings may provide important clues of likely etiology. Complaints of diarrheal disease are a commonly shared co-morbidity aspect in HIV/AIDS patients, in malignancy, in other intestinal parasitosis as severe trichuriasis, severe hookworm infection, balantidiasis and giardiasis, cystoisosporiasis, and alcoholism [30–33]. Gastrointestinal presentations of chronic *S. stercoralis* infection are non-specific and include diarrhea, nausea, anorexia, weight loss that may wax and wane before spontaneous resolution or proceed to hyperinfection and dissemination, most often in patients with impaired cell mediated immunity. Proper screening in high-risk patients like the ones described in this case-series, and before steroid treatment is mandatory as part of the differential diagnosis in order to guarantee prompt management.

Not all patients with strongyloidiasis had a report of eosinophilia in the peripheral blood, a condition significant and consistent attributed to this and other intestinal nematodes [34]. In 11 patients complete blood counts with differential counts was either not requested or annotation of results was omitted in the clinical file. We do not have a plausible explanation for this omission, except in two instances of laboratory infrastructure problems. Normal eosinophil level or no eosinophilia in nine patients have to be interpreted with caution, since no follow-up of cases was reported. Lack of peripheral eosinophilia in patients with strongyloidiasis carry a poor prognosis for the patient and may contribute to decrease suspicion of the infection [29]; among the eight autopsy cases from Costa Rica, only two presented with 6 % and 22 % eosinophilia, respectively [13].

There are no published studies conducted in any community or region of the country aimed at investigating the epidemiology of *S. stercoralis* infection; therefore it is not possible to identify risk factors for acquiring this infection. Nevertheless, we can say that in general patients were poor, lacked proper sanitary facilities, lacked schooling, most were adults, many came from rural areas and many members of a family shared inadequate housing [10, 11, 35, 36]. Since many countries in Latin America have a compromise to control neglected parasitic infections where strongyloidiasis is also common [2, 37], future investigations on the prevalence of soil transmitted helminths necessary for the implementation of coordinated preventive chemotherapy programs should include tested methods for *Strongyloides* larvae detection and identification through the application of coproparasitologic, serologic or molecular techniques appropriate for recovery and differentiation of this parasite [38].

To our knowledge none of the patients were further studied for signs or symptoms of hyper-infection or dissemination, no sputum, urine, duodenal aspirate or repeated stool samples were sent for larval detection. Only 10/35 received some anti-*Strongyloides* treatment with no follow-up exams for cure determination before hospital release. A review of original publications of *Strongyloides* infection in Indigenous Australian people [12] described as particular issues delayed diagnosis, inadequate knowledge, inadequate treatment and treatment dosages, lack of communication and lack of follow-up by health professionals. The authors highlighted the risks of *Strongyloides* infection confronted by rural population, children and immunosuppressed individuals and described a series of actions addressing barriers to control: development of reporting protocols, documentation of current infection sites, requirements of health professionals to have detailed information and education regarding strongyloidiasis, testing treatment initiatives in the community. Coordinated approaches and supported community initiatives could well result in eradication of endemic strongyloidiasis in Australia and why not? elsewhere. We hypothezise that a good share of the problem in Honduras mirrors the situation in Australia as it relates to deficient parasitology literacy among health personnel [39] translated in failure to recognize symptoms, coupled with inadequate knowledge for treatment and little or no interest in conducting community studies to define the parasitological problems locally. Another support for the need of sound parasitology training is reflected in a recent publication [40] from 15 different training programs world-wide of in-training physician assessing the knowledge in parasitic diseases and strongyloidiasis, comparing performance of residents from the United States of America (US) with residents from other countries. The evaluation focused on resident recognition and diagnostic recommendations. In answers regarding the need for parasite screening, US residents had poor recognition compared with international physicians in-training (9 % vs. 56 %, *p* = 0.001), respectively; 41 % were unable to name parasites causing pulmonary symptoms. Still, 44 % residents from developing countries were unaware of the potentially unwanted iatrogenic complications of strongyloidiasis and treatment protocols.

## Conclusions

Several important limitations in this study relate to insufficient availability of clinical data, missing follow-up of patients, poor patient management and weak laboratory methodology. However, we demonstrated that *S. stercoralis* infection represents a considerable problem in patients consulting at the UH in Honduras, physician awareness was very low and urgent action is required to improve training and knowledge of intestinal parasitic infections; the high burden of strongyloidiasis calls for regular inclusion of more specific techniques such as Baermann and/or Koga agar migration method [23, 24, 41] in all public health laboratories of Honduras [42] for the diagnostic work-up of patients, particularly those with persistent diarrhea.

## References

[1] Olsen A, Van Lieshout L, Marti H, Polderman T, Polman K, Steinmann P, Stothard R, Thybo S, Verweij JJ, Magnussen P. Strongyloidiasis - the most neglected of the neglected tropical diseases? Trans R Soc Trop Med Hyg. 2009;103:967–72. 10.1016/j.trstmh.2009.02.013 19328508

[2] Krolewiecki AJ, Lammie P, Jacobson J, Gabrielli AF, Levecke B, Socias E (2013). A public health response against *Strongyloides stercoralis*: time to look at soil-transmitted helminthiasis in full. PLoS Negl Trop Dis.

[3] Jorgensen T, Montresor A, Savioli L (1996). Effectively controlling strongyloidiasis. Parasitol Today.

[4] Bisoffi Z, Buonfrate D, Montresor A, Requena-Méndez A, Muñoz J, Krolewiecki AJ, Gotuzzo E, Mena MM, Chiodini PL, Anselmi M, Moreira J, Albonico M. *Strongyloides stercoralis*: A Plea for Action. PLoS Negl Trop Dis. 2013;7(5), e2214. 10.1371/journal.pntd.0002214 PMC3649953 23675546

[5] Schär F, Trostdorf U, Giardina F, Khieu V, Muth S, Marti H, Vounatsou P, Odermatt P. *Strongyloides stercoralis*: global distribution and risk factors. PLoS Negl Trop Dis. 2014;7(7), e2288. 10.1371/journal.pntd.0002288 PMC3708837 23875033

[6] Hotez P, Kamath A (2009). Neglected tropical diseases in Sub-Saharan Africa: review of their prevalence, distribution, and disease burden. PLoS Negl Trop Dis.

[7] Becker SL, Sieto B, Silué KD, Adjossan L, Koné S, Hatz C (2011). Diagnosis, clinical features, and self-reported morbidity of *Strongyloides stercoralis* and hookworm infection in a co-endemic setting. PLoS Negl Trop Dis.

[8] Posey DL, Brian G, Blackburn BG, Michelle Weinberg M, Flagg EW, Ortega L, Wilson M, Secor WE, Sanders-Lewis K, Won K, Maguire JH. Schistosomiasis and strongyloidiasis among African refugees. Clin Infec Dis. 2007;45:1310–5. 10.1086/522529 17968826

[9] Keystone JS (2007). Can one afford not to screen for parasites in high-risk immigrant populations?. Clin Inf Dis.

[10] Khieu V, Schärr F, Forrer A, Hattendorf J, Marti H, Duong S, Vounatsou P, Sinuon Muth S, Odermatt P. High prevalence and spatial distribution of *Strongyloides stercoralis* in rural Cambodia. PLoS Negl Trop Dis. 2014;8(6):e2854. 10.1371/journal.pntd.0002854 PMC4055527 24921627

[11] Wang C, Xu J, Zhou X, Li J, Yan G, James AA, Chen X. Review: Strongyloidiasis: An emerging infectious disease in China. Am J Trop Med Hyg. 2013;88(3):420–25. 10.4269/ajtmh.12-0596 PMC3592519 23468357

[12] Miller A, Smith ML, Judd JA, Speare R (2014). *Strongyloides stercoralis*: systematic review of barriers to controlling strongyloidiasis for Australian Indigenous Communities. PLoS Negl Trop Dis.

[13] Arroyo R, Troper L, Vargas G (1985). Fatal strongyloidiasis. An eight autopsies case study. Patol.

[14] Institute for Infectious Diseases and Parasitology and Panamerican Health Organization/Honduras (2009). Manual for Management of Priority Parasitic Infections in Honduras. 2ª. Ed.

[15] Prendki V, Fenaux P, Durand R, Thellier M, Bouchaud O (2011). Strongyloidiasis in man 75 years after initial exposure. Emerg Infect Dis.

[16] Davidson RA (1992). Infection due to *Strongyloides stercoralis* in patients with pulmonary disease. Southern Med J.

[17] Croker C, Reporter R, Redelings M, Mascola L (2010). Strongyloidiasis-related deaths in the United States, 1991–2006. Am J Trop Med Hyg.

[18] Buonfrate D, Requena-Mendez A, Angheben A, Muñoz J, Gobbi F, Van Den Ende J, Bisoffi Z. Severe strongyloidiasis: a systematic review of case reports. BMC Infect Dis. 2013;13:78–87. 10.1186/1471-2334-13-78 PMC3598958 23394259

[19] Fischer JN (2015). *Strongyloides* ‘larva currens’following high dose dexamethasone for upper airway burns: A case report and brief review of the literature. Trop Med Surg.

[20] Fardet L, Généreau T, Poirot JL, Guidet B, Kettaneh A, Cabane J. Severe strongyloidiasis in corticosteroid treated patients: case series and literature review. J Infect. 2007:54-18-27. 10.1016/j.jinf.2006.01.016 16533536

[21] Sousa Braza A, Ferreira de Andrade CA, Henrique da Motad LM, Bezerra Luna Limae CM. Recommendations from the Brazilian Society of Rheumatology on the diagnosis and treatment of intestinal parasitic infections in patients with autoimmune rheumatic disorders. Rev Brasileira Reumatol 2015. http://dx.doi.org/10.1016/j.rbre.2014.10.010 10.1016/j.rbr.2014.10.010 25583002

[22] Gotuzzo E, Terashima A, Alvarez H, Tello R, Infante R, Watts DM, Freedman DO. *Strongyloides stercoralis* hyperinfection associated with human T cell lymphotropic virus type-1 infection in Peru. Am J Trop Med Hyg. 1999;60(1):146–9. 10.4269/ajtmh.1999.60.146 9988339

[23] Dreyer G, Fernández-Silva E, Alves S, Rocha A, Albuquerque R, Addiss D (1996). Patterns of detection of *Strongyloides stercoralis* in stool specimens: implications for diagnosis and clinical trials. J Clin Microbiol.

[24] Inês EJ, Souza JN, Santos RC, Souza ES, Santos FL, Silva MLS, Silva MP, Teixeira MCA, Soares NM. Efficacy of parasitological methods for the diagnosis of *Strongyloides stercoralis* and hookworm in faecal specimens. Acta Trop. 2011;120:206–10. 10.1016/j.actatropica.2011.08.010 21896267

[25] Yori PP, Kosek M, Gilman RH, Córdova J, Bern C, Banda Chávez C, Paredes Olortegui M, Montalvan , Meza Sánchez G, Worthen B, Worthen J, Leung F Vidal Ore. Seroepidemiology of strongyloidiasis in the Peruvian amazon. Am J Trop Med Hyg. 2006;74:97–102. PMC1483914 16407351

[26] Sitta RB, Malta FM, Pinho JR, Chieffi PP, Gryschek RC, Paula FM (2014). Conventional PCR for molecular diagnosis of human strongyloidiasis. Parasitol.

[27] Kaminsky RG (2005). Disseminated strongyloidiasis in a patient with AIDS, Honduras. Rev Méd Hondur.

[28] Kaminsky RG (1993). Evaluation of three methods for the identification of *Strongyloides stercoralis* infection. J Parasitol.

[29] Marcos LA, Terashima A, Canales M, Gotuzzo E (2011). Update on Strongyloidiasis in the immunocompromised host. Curr Infect Dis Rep.

[30] Keiser PB, Nutman TB (2004). *Strongyloides stercoralis* in the immunocompromised population. Clin Microbiol Rev.

[31] Sidhartha JM, Mohan BM, Penchalaiah M, Reddy LVPK (2015). Strongyloidiasis after corticosteroid therapy: a case report. Int J Case Rep Images.

[32] Marques CC, Zago-Gómes MP, Sandoval Goncalves C, Lima Pereira FE (2010). Alcoholism and *Strongyloides stercoralis*: daily ethanol ingestion has positive correlation with the frequency of *Strongyloides* larvae in stools. PLoS Negl Trop Dis.

[33] Siegel MO, Simon GL (2012). Is human immunodeficiency virus infection a risk factor for *Strongyloides stercoralis* hyperinfection and dissemination?. PLoS Negl Trop Dis.

[34] Kaminsky RG, Soto RJ, Campa A, Baum M (2000). Intestinal parasitic infections and eosinophilia in a human immunodeficiency virus positive population in Honduras. Mem Inst Oswaldo Cruz Rio de Janeiro..

[35] Paula FM, Costa-Cruz JM (2011). Epidemiological aspects of strongyloidiasis in Brazil. Parasitol.

[36] Lindo JF, Robinson RD, Terry SI, Vogel P, Gam AA, Neva FA, Bundy DAP. Age-prevalence and household clustering of *Strongyloides stercoralis* infection in Jamaica. Parasitol. 1995;110:97–102. 10.1017/s0031182000081099 7845718

[37] Ault SK (2007). Pan American Health Organization’s Regional Strategic Framework for addressing neglected diseases in neglected populations in Latin America and the Caribbean. Mem Inst Oswaldo Cruz, Rio de Janeiro.

[38] Ützinger J, Becker SL, Knopp S, Blum J, Neumayr AL, Keiser J, Hatz CF. Neglected tropical diseases: diagnosis, clinical management, treatment and control. Swiss Med Wkly. 2012;142:w3727. 10.4414/smw.2012.13727 23180107

[39] Kaminsky R (1992). Teaching of medical parasitology in Honduras. An answer to national needs?. Med Clin.

[40] Boulware DR, Stauffer WM, Hendel-Paterson BR, Lopes Rocha JL, Chee-Seong Seet R, Summer AP, Nield LS, Supparatpinyo K, Chaiwarith R, Walker PF. Maltreatment of *Strongyloides* infection: Case series and worldwide physicians-in-training survey. Am J Med. 2007;120(6):545e1–8. 10.1016/j.amjmed.2006.05.072 PMC1950578 17524758

[41] Hirata T, Nakamura H, Kinjo N, Hokama A, Kinjo F, Yamane N, Fujita J. Increased detection rate of *Strongyloides stercoralis* by repeated stool examinations using the agar plate culture method. Am J Trop Med Hyg. 2007;77:683–4. 17978071

[42] García JA, López W, Alger J, Matute ML, Kaminsky RG (2014). Parasitology diagnosis in private and public health laboratories in Honduras: capacity to deliver?. Rev Med Hondur.

